# Targeting p53 by small molecules in hematological malignancies

**DOI:** 10.1186/1756-8722-6-23

**Published:** 2013-03-27

**Authors:** Manujendra N Saha, Lugui Qiu, Hong Chang

**Affiliations:** 1 Division of Molecular and Cellular Biology, Toronto General Research Institute, Toronto, Canada; 2 Dept. of Laboratory Medicine & Pathobiology, University of Toronto, Toronto, Canada; 3 Institute of Hematology and Blood Disease Hospital, Chinese Academy of Medical Sciences and Peking Union Medical College, Tianjin, China; 4 Dept. of Laboratory Hematology and Medical Oncology, University Health Network, Toronto, Ontario, Canada

**Keywords:** Hematological malignancies, Leukemia, Lymphoma, Myeloma, p53, Nutlin, RITA, PRIMA-1, MIRA-1, Apoptosis

## Abstract

p53 is a powerful tumor suppressor and is an attractive cancer therapeutic target. A breakthrough in cancer research came from the discovery of the drugs which are capable of reactivating p53 function. Most anti-cancer agents, from traditional chemo- and radiation therapies to more recently developed non-peptide small molecules exert their effects by enhancing the anti-proliferative activities of p53. Small molecules such as nutlin, RITA, and PRIMA-1 that can activate p53 have shown their anti-tumor effects in different types of hematological malignancies. Importantly, nutlin and PRIMA-1 have successfully reached the stage of phase I/II clinical trials in at least one type of hematological cancer. Thus, the pharmacological activation of p53 by these small molecules has a major clinical impact on prognostic use and targeted drug design. In the current review, we present the recent achievements in p53 research using small molecules in hematological malignancies. Anticancer activity of different classes of compounds targeting the p53 signaling pathway and their mechanism of action are discussed. In addition, we discuss how p53 tumor suppressor protein holds promise as a drug target for recent and future novel therapies in these diseases.

## Introduction

p53, ‘guardian of the genome’, was the first tumor suppressor gene to be identified in 1979. p53 functions to eliminate and inhibit the proliferation of abnormal cells, thereby preventing tumor development [1-4]. The human p53 gene is located on chromosome 17p and consists of 11 exons and 10 introns [5]. The central role of p53 in the cells suggests that the loss of p53 function may have severe consequences. The p53 function is lost in an estimated 50% of human cancers by mutations or deletions in p53 gene [6]. The frequency of mutation in p53 is, however, lower in hematological cancers than in solid tumors [7-12]. For example, TP53 is mutated in 10-20% of cases of chronic lymphocytic leukemia (CLL) [7,8], 3-8% of cases of acute myeloid leukemia (AML) [8], less than 3% in acute lymphoblastic leukemia (ALL) [9], and 10-12% cases of multiple myeloma (MM) [10-12]. Importantly, in hematological malignancies, deletion/mutation of p53 is associated with high risk i.e., more aggressive disease, worse overall survival and resistance to therapies [7-13]. In the presence of wild type p53 other mechanisms may affect the expression and activity of p53 which include elevated expression of the negative regulators of p53, murine double minute 2 (MDM2) [14-20]. MDM2 is transcriptionally activated by the binding of p53 to a p53-responsive element within its gene. It then binds to the N-terminal region of p53, thereby preventing p53 from interacting with the transcriptional machinery and inducing its degradation [15,18,20-23].

**Figure 1:**
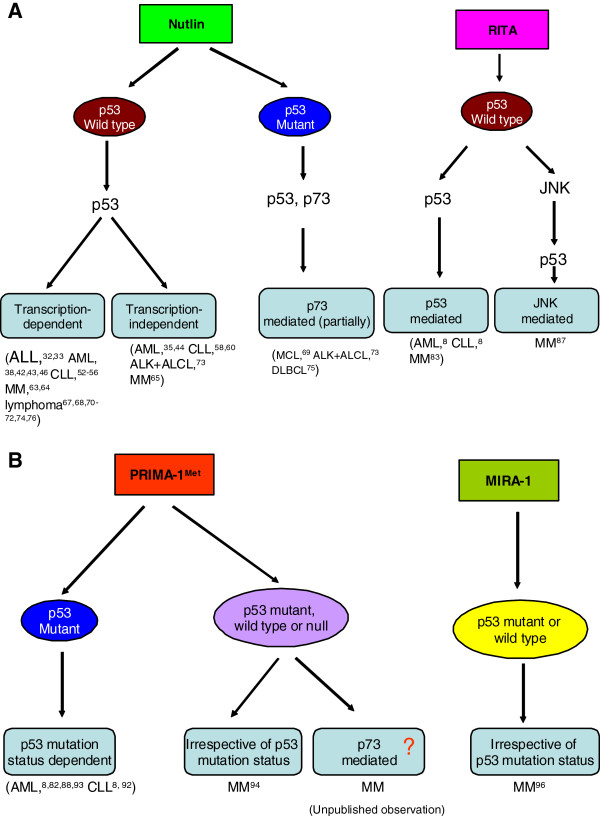
**Strategies for induction of apoptosis by small molecules targeting p53 in hematological malignancies. (A)** Nutlin-induced apoptosis in cells harboring wild type p53 can be mediated by p53-transcription-dependent and/or -independent pathways. In mutant p53 cell types, nutlin-induced apoptosis can be mediated by activation of p53 and/or p73. Small molecule RITA activates wild type p53 for the induction of apoptosis in different types of hematological malignancies including AML, CLL, and MM. However, in MM, RITA-induced activation of p53 can be mediated by either direct activation of p53 or through activation of the JNK signaling pathway. **(B)** PRIMA-1^Met^-induced apoptosis in AML and CLL cells has been shown to be p53 mutation status dependent. However, it can induce apoptosis in MM cells irrespective of p53 status or even in the absence of p53. The apoptosis induction by PRIMA-1^Met^ in MM cells in the presence or absence of p53 as suggested by us is mediated by activation of p73 signaling. Small molecule MIRA-1, which has originally been described as a mutant p53 activator is shown to induce apoptosis of MM cells independent of p53 mutation status, i.e., it can induce apoptosis in MM cells harboring either wild type or mutant p53.

There are evidences that many anti-cancer drugs induce apoptosis through multiple pathways that are at least in part dependent upon p53 activation [16-23]. Attempts have been made to develop strategies based on the small molecules to specifically modulate the activity of p53 proteins. These approaches can be classified into two categories: those that aim at modulating the activity of wild-type p53 (Figure 1A) and those that aim at restoring wild-type functions in cells expressing mutant p53 (Figure 1B). The small molecules have been identified by either cellular or protein assays [20-23]. The cellular approach involves screening to identify compounds which can cause tumor cell death. An advantage of this approach is that the compounds identified e.g., nutlin, RITA (Reactivation of p53 and induction of tumor cell apoptosis) and PRIMA-1 (p53 reactivation and induction of massive apoptosis) have a desired biological outcome such as apoptosis and rarely display genotoxicity [24-27]. However, it is difficult to elucidate their exact molecular mechanism for apoptosis. On the other hand, a protein based approach can identify compounds e.g., CP-31398 that directly affect a target protein. But the compounds may be toxic or may not have adequate bioavailability [27-31]. A number of small molecules with activities fitting within these two categories have been identified and some of those have already progressed to advanced preclinical development or early-phase (phase I/II) clinical trials (Table 1). In this review we will describe all of these aspects of targeting p53 in hematological malignancies.

**Table 1:** Small molecules used for targeting p53 in various hematoloigcal malignancies

**Hematological malignancy**	**Small molecule**	**Potential target**
**Leukemia**		
ALL	Nutlin	MDM2^32-34^
	RITA	p53^82^
	JNJ-26854165	p53, E2F1^99^
AML	*Nutlin	MDM2^35,38,42,43,46^
	MI-219	MDM2^48^
	RITA	p53^8^
	*PRIMA-1	p53^8,82,88,93^
	JNJ-26854165	p53, E2F1^99^
CLL	Nutlin	MDM2^54-56,58-60^
	RITA	p53^8^
	PRIMA-1	p53^8,92^
CML	Nutlin	MDM2^61,62^
	MI-219	MDM2^61^
**Lymphoma**		
HL	Nutlin	MDM2^67,68^
MCL	Nutlin	MDM2^69-72^
Birkitt’s lymphoma	Nutlin	MDM2^76^
B Cell lymphoma	MI-219	MDM2^74^
Follicular lymphoma	MI-319	MDM2^78^
**Myeloma**	Nutlin	MDM2^63-65^
	RITA	p53^84^, JNK^87^
	PRIMA-1^Met^	p53, p73^94^
	MIRA-1	p53^96^
	Halofuginone	p53^97^

*in phase I/II clinical trials.^91,101^.

### Anti-tumor activities of nutlin in hematological malignancies

Of the small molecules that inhibit the protein–protein interaction between p53 and MDM2, the first reported was nutlins [24]. Nutlin is a nongenotoxic compound which binds to the p53-binding pocket in the MDM2 protein, thus releasing p53 from the negative control of MDM2 leading to effective p53 stabilization and activation in cancer cells with wild type but not mutant or deleted p53 [24,30,31]. Since its discovery, nutlin has been one of the most investigated small molecules in the field of cancer therapy and has shown considerable promise in this area. In pre-clinical studies, nutlin alone or in combination with chemotherapeutic drugs has displayed increasing potential for the treatment of blood malignancies [32-79].

#### Preclinical studies of nutlin in ALL and AML

Nutlin induced cytotoxic and apoptotic response in both ALL [32-34] and AML [35-49] cells including the cell lines and/or patient samples with little effect on normal CD34+ hematopoietic progenitor cells [35]. Nutlin-mediated killing of ALL cells harboring wild type p53 and over-expressing MDM2 is clinically very much significant since all patients with leukemic cells over-expressing MDM2 are usually resistant to conventional therapy and have a poor prognosis [32]. Nutlin-induced apoptosis in AML cells can be mediated by both p53-transcription-dependent and -independent pathways [35] (Figure 1A). Moreover, nutlin displayed synergistic responses in AML cells with several anti-leukemic agents including a Bcl2 antagonist (ABT-737) [36], MEK inhibitors (PD98059) [37] and (AZD6244) [38], recombinant TRAIL [39,40]; FI-700, an FLT3 inhibitor [41], a vitamin D metabolite (1-25D) [42], HDAC inhibitor (valporic acid) [43], PI3K/mTOR inhibitor (PI-103) [44], perifosine (an Akt inhibitor) [49], and sorafenib, a second generation protein kinase inhibitor which is in phase I clinical trial [45].

#### Cytotoxic response of nutlin in CLL and chronic myeloid leukemia (CML)

Studies have shown that selective p53 activation with nutlin variably induced apoptosis in both low- and high-risk subtypes of B-CLL [50-60] and CML [61,62] patient cells. Notably, nutlin induced cytotoxicity toward B-CLL cells at concentrations that were less toxic toward normal B lymphocytes, peripheral blood mononuclear cells (PBMCs), and bone marrow (BM) hematopoietic progenitor cells [54]. In addition to conventional p53 transcriptional pathway, nutlin also induced apoptosis in CLL cells by mitochondrial pathway (Figure 1A) [58,60].

#### Anti-myeloma activity of nutlin

We and others have demonstrated potent anti-myeloma activity of nutlin by molecular and functional analysis of the p53 pathway in MM cell lines, primary MM patient samples and in the cells of the bone marrow microenvironment [63-65]. We explored the molecular mechanisms for nutlin-induced apoptosis in MM cells and provided the evidence for association of both p53-transcription-dependent and -independent pathways (Figure 1A) [65]. Our study supports the concept that the transcriptional and mitochondrial functions of p53 are equally important for nutlin-triggered apoptosis, perhaps depending on cancer cell types and their local micro-environments [65,66].

#### Anti-tumor activity of nutlin in lymphoma and adult T-cell leukemia (ATL)

Among different lymphoma models, nutlin has been shown effective in inducing wild type p53-dependent apoptosis of Hodgkin’s lymphoma (HL) [67,68], mantle cell lymphoma (MCL) [69-72], ALK-positive anaplastic large cell lymphoma (ALCL) [73], B-cell lymphoma (BCL) [74,75], Burkitt’s and follicular lymphoma [76-78], and adult T cell leukemia [79]. Interestingly, when combined with geldanamycin (an HSP90 inhibitor) nutlin exerted its apoptotic activity in both p53 wild type and mutant HL cells since geldanamycin-induced apoptosis in HL cells was p53-independent [68]. MDM2 inhibition by nutlin successfully induced intrinsic mitochondrial apoptotic activation through increased expression of Noxa in refractory MCL cells, which had limited sensitivity to bortezomib alone. The Nutlin/bortezomib combination enhanced Noxa protein expression in mutant p53 cells but not in wild type p53 MCL cells [71]. Similar to our observations in MM [65], nutlin-induced apoptosis in ALCL cells involved both p53-mediated transcriptional and non-transcriptional mechanisms [71,73]. Recently, by both *in vitro* and *in vivo* evidence Drakos et al. demonstrated that nutlin induced cell cycle arrest and apoptosis in DLBCL cells with functional p53, t(14;18)(q32;q21) translocation, and Bcl2 over-expression [75]. Importantly, combined treatment with nutlin and doxorubicin synergistically inhibited the growth of ALCL or DLBCL cells harboring either wild type or mutant p53 [73,75]. These studies also demonstrated that nutlin induced increased expression of p73 in MCL, ALCL, or BCL cells harboring mutant p53 [72,73,75]*.* Activation of p53 by nutlin resulted in both cellular senescence and apoptosis in ATL-related cell lines harboring wild type p53 suggesting that cellular senescence might be an important event in p53-dependent cell death in ATL cells [79].

### Targeting p53 by RITA

RITA (also known as NSC 652287) was identified through a screening assay based on a library. Upon binding to p53, RITA reactivates it and induces apoptosis by disrupting the interaction with MDM2 [25,30]. Although the IC_50_ values for RITA vary depending on tumor cell type, growth inhibition is clearly more effective in wild type p53-expressing cells [25,30,80-87].

#### Anti-leukemic activity of RITA

Among hematological malignancies, anti-tumor activity of RITA was first described in a panel of CLL and AML patient samples [8]. This study described a constitutive activation of the p53 pathway leading to cell cycle arrest and apoptosis by RITA in CLL and AML cells harboring wild type p53 [8]. However, RITA acted synergistically with fludarabine in CLL cells irrespective of p53 status and with PRIMA-1 in AML cells with or without p53 deletion [8].

#### Anti-tumor activity of RITA in MM and MCL

Anti-tumor activity of RITA in MM cells was first described by our group in 2010 [83]. Our *in vitro* studies demonstrated that RITA displayed potent anti-myeloma activities in MM cells harboring wild type p53 without killing normal cells [83]. The *in vitro* observation was further confirmed in xenograft mouse model of MM where we have demonstrated significant inhibition of tumor growth and prolongation of survival in mice bearing MM tumors [84,85]. RITA was initially thought to bind with amino terminal domain of p53, inducing a conformational change of the protein and increasing its half life and its accumulation in tumor cells. However, the results of a recent nuclear magnetic resonance (NMR) study indicated that RITA might affect p53 function by other mechanisms, not involving binding to its N-terminal, such as interaction with other binding proteins and cofactors [86]. In keeping with this theory, most recently we provided the evidence that RITA targeted c-Jun N-terminal Kinase (JNK) for the induction of apoptosis in MM cells suggesting that RITA might function as a multi-target molecule [87] (Figure 1A). Further studies are needed to identify the specific binding targets for RITA.

Interestingly, study by Jones et al. provided the evidence that continuous exposure of MCL and MM models to two different MDM2 inhibitors MI-63 and nutlin resulted in p53 point mutations as a mechanism of acquired drug resistance, and that RITA might overcome this resistance by restoring p53 function [81]. This study, therefore, suggests simultaneous restoration of p53 function and MDM2 inhibition as a rational strategy for clinical translation. In support of this, we showed that RITA in combination with nutlin displayed synergistic cytotoxic response in MM cells [83]. The combination of RITA with MI-63 resulted in synergistic response in both MCL and MM cell lines resistant to MI-63 or nutlin [81]. In addition, our studies showed that RITA exerted synergistic response in combination with current chemotherapeutic agents such as doxorubicin or dexamethasone or with the JNK activator 2-Cyano-3,12-dioxooleana-1,9-dien-28 oic Acid (CDDO) [87].

### Other small molecules targeting p53-MDM2 interaction

Among the other small molecule MDM2 inhibitors examined in hematological malignancies are MI-63, MI-219, and MI-319 [48,61,69,74,78,81]. MI-63 showed synergistic response with gemcitabine in MCL cells [69]. A most recent study demonstrated that MM or MCL cell lines resistant to either MI-63 or nutlin showed cross-resistant to the other, and were less sensitive to bortezomib, doxorubicin, cisplatin, and melphalan, but not to RITA. Exposure to resistant cells (MM and MCL) to RITA induced cell cycle arrest and activation of p53-transcriptional targets, supporting a restoration of p53 activity. Combination of RITA and MI-63 showed re-sensitization of resistant MM or MCL cells to MI-63 [81]. Similar to nutlin, MI-219 binds to MDM2 with a high affinity, activated the p53 pathway and selectively inhibited cell growth in cancer cell lines with wild type p53. Anti-tumor activities of MI-219 have been described in AML [48], CML [61], and B-cell lymphoma [74]. Since MI-219/319 achieved an excellent oral bioavailability, it was tested in mouse xenograft models of human follicular lymphoma where MI-319 treatment resulted in inhibition of tumor growth and prolongation of survival [78]. These results suggest that MI-63, MI-219 or MI-319 may be considered as a promising cancer therapy with possible future clinical applications.

### Restoration of wild type conformation of mutant p53

Targeting mutant p53 by small molecules appears as an even greater challenge than activating wild type p53 in a tumor cells. Various strategies for reconstitution of wild-type p53 function in tumors have been successfully developed and some have even reached the clinic. Screening approaches have led to the identification of small molecules which can restore p53 function in tumor cells [21-23,26,27]. Here we will focus on two initially reported mutant p53 reactivating drugs (PRIMA-1/PRIMA-1^Met^, and MIRA-1) and describe their anti-tumor activities in hematological malignancies.

### PRIMA-1

PRIMA-1 is a low-molecular weight compound that can restore wild-type conformation of mutant p53 and specific DNA binding, consequently triggering apoptosis in tumour cells carrying mutant p53 [21,26,27,88]. Both unfolded mutant p53 and unfolded wild type p53 can be refolded by PRIMA-1 [89]. The identification of PRIMA-1 as an anti-tumor agent goes back to 2002 in a Swedish Lab where it was found that PRIMA-1 had preferential growth inhibitory activity on different type of human cancer cell lines carrying mutant p53 [21,26,27,90]. This distinguishes PRIMA-1 from anticancer drugs commonly used in treatment of malignant disease [90]. It takes about 10 years for this drug to come into its successful clinical trial in 2012 [91] in a hope to make it a promising anti-cancer drug.

#### Anti-leukemic activity of PRIMA-1/ PRIMA-1^Met^

PRIMA-1 was initially tested on 60 human tumor cell lines including lymphoma tumor cell lines [26,90]. A few years later, Nahi et al. reported the effect of PRIMA-1 in leukemic cells from CLL and AML patients with or without p53 deletion [88,92]. There were no obvious differences in cytotoxic response of PRIMA-1 between hemizygous p53 deleted and non-deleted CLL samples [92]. However, PRIMA-1 was more cytotoxic to AML cells with hemizygous p53 deletion/mutation [88]. Several studies including ours showed that normal hematopoietic cells were relatively resistant to PRIMA-1 in the concentrations used to kill tumor cells [92-94]. PRIMA-1 has been shown to display synergistic or additive response in combination with fludarabine in CLL [92] and AML [93]. The methylated analog of PRIMA-1, PRIMA-1^Met^, has even greater potency [27], leading to its development as a candidate therapeutic drug under the code name APR246 which is in phase I/II clinical trial [91].

#### Anti-myeloma activity of PRIMA-1^Met^

The therapeutic concept is that PRIMA-1^Met^ may selectively rescue mutant p53 and induce apoptosis in cancer cells, leaving wild-type p53 in normal cells mostly unaffected [26]. However, so far there is little information on how PRIMA-1^Met^ affects p53 in cancer cells with no mutation. At first sight, PRIMA-1^Met^ activates wild type p53 appears to contradict claims that the drug is specific for mutant p53 [26,27]. However, there are good biochemical reasons to propose that the drug operates as an all-around rescuer of inactive p53, independent of p53 mutation status. In some cancer cells, p53 protein activity may be disrupted by other mechanisms with functional consequences equivalent to mutation. How such a functionally disrupted p53 may react to PRIMA-1^Met^ is unknown. In the meantime, the recent studies including our preliminary results added a new dimension to the potential of PRIMA-1^Met^ as a therapeutic drug by showing that it induced apoptosis in cells bearing wild type p53 or even in the absence of p53 (Figure 1B) [92-94]. Thus, the therapeutic usage of PRIMA-1^Met^ may be extended for treatment of the vast majority of tumors with a broader spectrum.

### Anti-tumor activity of MIRA-1

Using a similar method as described for PRIMA-1, Bykov et al. identified a novel class of molecules that are structurally distinct from PRIMA-1. MIRA-1, the first of these molecules to be tested, induces apoptosis in cells containing mutant p53 with even higher potency than PRIMA-1 [95]. The reactivation of mutant p53 by MIRA-1 has been demonstrated by studies revealing the induction of expression p53-target genes such as p21, MDM2 and Puma in solid tumor cell lines. Therefore MIRA-1 and its structural analogs are postulated to act by shifting the equilibrium between the native and unfolded conformations of p53 toward the native conformation, leading to the restoration of p53-mediated transactivation of target genes and the induction of p53-dependent apoptosis. We have examined anti-tumor activities of MIRA-1 in MM cell lines and patients samples. The results of our preliminary studies showed that anti-myeloma activity of MIRA-1 was independent of p53 status (Figure 1B) [96].

### Other miscellaneous small molecules targeting p53 in hematological malignancies

Recent *in vitro* and *in vivo* studies by Leiba et al. showed that the small molecule halofuginone hydrobromide (HF), a synthetic derivative of quinazoline alkaloid, triggered growth inhibition in both MM cell lines and patient samples. In addition, HF enhanced cytotoxicity of conventional (melphalan, dexamethasone, and doxorubicin) and novel anti-MM (such as lenalidomide) agents [97]. Similar to nutlin, CLL cells with p53 deletion was less sensitive to the small molecule Bcl2 inhibitor ABT-737 than the cells without p53 deletion [98]. JNJ-26854165, a tryptamine derivative has been shown to block the proteasomal degradation of p53 and induce apoptosis in both wild type and mutant p53 expressing leukemia cell lines [99]. Due to its broad anti-tumour activity, JNJ-26854165 is being assessed in a phase I trial as an oral agent for advanced solid tumors [96]. An Aurora kinase inhibitor MLN8237 has been shown effective in killing myeloma cells *in vitro* and *in vivo*[100].

### Clinical trials with small molecules targeting p53 in hematological malignancies

The first MDM2 inhibitor that entered clinical development is RG7112 (RO5045337), a member of the nutlin family, from Hoffmann-La Roche (*clinicaltrials.gov*; identifiers: NCT01164033, NCT01143740, NCT00623870, and NCT00559533). RG7112 was given to patients with acute or chronic relapsing or refractory leukemia orally every day for 10 days followed by 18 days of rest. Preliminary clinical data indicated that RG7112 appeared to be well tolerated in patients and showed initial evidence of clinical activity and a mechanism of action consistent with targeting the p53-MDM2 interaction [101].

PRIMA-1^Met^**(**APR-246) has recently been tested on humans in a phase I/II study, which was conducted on 22 patients with advanced blood or prostate cancer. The patients received daily infusions of APR-246 for four days. Analysis of the cancer cells taken before and after treatment showed activation of the p53 leading to apoptosis of cancer cells. Ten patients could be evaluated as regards the development of their cancer, and in two of them there were signs of tumor regression [91].

Since tumor cells can acquire resistance to MDM2 inhibitors or current therapeutic agents through p53 mutation it is important to prevent the development of drug resistance and secondary cancer. The combination approaches has been proved successful in this regard as well as to reduce the side-effects of the drugs. A number of studies have demonstrated that nutlin may be used not just as a single agent but also in combination with other anti-cancer agents to achieve better anti-tumor activity than alone. For example, *ex vivo* experiments using patient tumor samples have shown that nutlin synergizes with doxorubicin, chlorambucil, and fludarabine in B-CLL [30,50,51,55,62]; with doxorubicin and cytosine arabinoside in AML [30,35,36,42]; with doxorubicin and etoposide in HL [67,68]; with melphalan, bortezomib, and lexatumumab (a DR5 agonist) in MM [63,65,102,103]; and with bortezomib in MCL [71], and with doxorubicin in ALCL and DLBCL [73,75]. Moreover, synergistic response of fludarabine with nutlin, RITA or PRIMA-1^Met^ in CLL and/or AML is clinically significant because treatment with fludarabine has been shown to increase the complete remission rate, enhance progression-free survival, and increase the median duration of the clinical response as compared with single therapy [45,56,58]. Importantly, nutlin in combination with bortezomib synergistically contributes to apoptosis induction not only in wild type-p53-possessing MCL cells, but also in MCL with known negative prognostic factors that include p53 mutation, and bortezomib resistance [71]. These findings indicate potential therapeutic efficacy of the small molecules in combination with current chemotherapeutics for the treatment of chemorefractory hematological malignancies.

### Conclusions and future directions

Although many of the cellular effects of the described small molecules require the presence of p53, the evidence of some p53-independent effects suggests that p53 may not be its only target. For example, the effect of nutlins initially seemed to be limited to cells harboring wild type p53, subsequent research revealed that nutlin also exerted its anti-cancer activities in p53-negative and p53-mutant human tumor cells through different mechanisms including activation of E2F1 or p73 in different types of cancers including hematological malignancies [48,72,104,105]. Similarly, the biological activity of RITA may be mediated by additional, still unknown, biochemical mechanisms. The global alteration of gene-expression profile rather than merely p53 targets following treatment of PRIMA-1^Met^ suggests other pathways may exist in PRIMA-1^Met^-induced cell death in MM cells [94]. There is also evidence that PRIMA-1^Met^ can stabilize p53 family members in solid tumors as well as in MM and this may be part of its mechanism of action in mutant p53-expressing tumors [106,107]. Although the exact molecular mechanisms remain unclear, it is possible that PRIMA-1^Met^ can release p73 from an inactive complex with mutant p53 (our unpublished observation). Thus, at least in certain conditions, targeting of p73 or p63 might be an interesting approach to interfere with alternative tumor suppressor pathways [107]. Identifying the potent and selective target(s) for these small molecules will not only be important for understanding the precise mechanisms of the action of the drugs but also provide the basis for improved drug design to preferentially kill cancer cells with only a limited toxicity towards normal cells.

## Competing interests

The authors declare that they have no competing interests.

## Authors’ contribution

MNS and HC carried out the primary literature search and wrote the paper. LQ participated in the discussion and editing the manuscript. All authors read and approved the final manuscript.

## References

[B1] Brown CJ Lain S Verma CS Fersht AR Lane DP Awakening guardian angels: drugging the p53 pathway Nat Rev Cancer 2009 9 862 873 10.1038/nrc2763 19935675

[B2] Meulmeester E Jochemsen AG p53: a guide to apoptosis Curr Cancer Drug Targets 2008 8 87 97 10.2174/156800908783769337 18336191

[B3] Lim YP Lim TT Chan YL Song AC Yeo BH Vojtesek B Coomber D Rajagopal G Lane D The p53 knowledgebase: an integrated information resource for p53 research Oncogene 2007 26 1517 1521 10.1038/sj.onc.1209952 16953220

[B4] Gomez-Lazaro M Fernandez-Gomez FJ Jordán J p53: twenty five years understanding the mechanism of genome protection J Physiol Biochem 2004 60 287 307 10.1007/BF03167075 15957248

[B5] Lamb P Crawford L Characterization of the human p53 gene Mol Cell Biol 1986 6 1379 1385 2946935 10.1128/mcb.6.5.1379-1385.1986 PMC367661

[B6] Soussi T Dehouche K Beroud C p53 website and analysis of p53 gene mutations in human cancer: forging a link between epidemiology and carcinogenesis Hum Mutat 2000 2 105 213 10612830 10.1002/(SICI)1098-1004(200001)15:1<105::AID-HUMU19>3.0.CO;2-G

[B7] Pekova S Mazal O Cmejla R Hardekopf DW Plachy R Zejskova L Haugvicova R Jancuskova T Karas M Koza V A comprehensive study of TP53 mutations in chronic lymphocytic leukemia: Analysis of 1287 diagnostic and 1148 follow-up CLL samples Leuk Res 2011 35 889 898 10.1016/j.leukres.2010.12.016 21232794

[B8] Nahi H Selivanova G Lehmann S Möllgård L Bengtzen S Concha H Svensson A Wiman KG Merup M Paul C Mutated and non-mutated TP53 as targets in the treatment of leukaemia Br J Haematol 2008 141 445 453 10.1111/j.1365-2141.2008.07046.x 18341636

[B9] Agirre X Novo FJ Calasanz MJ Larráyoz MJ Lahortiga I Valgañón M García-Delgado M Vizmanos JL TP53 is frequently altered by methylation, mutation, and/or deletion in acute lymphoblastic leukaemia Mol Carcinog 2003 38 201 208 10.1002/mc.10159 14639659

[B10] Avet-Loiseau H Li JY Godon C Morineau N Daviet A Harousseau JL Facon T Bataille R p53 deletion is not a frequent event in multiple myeloma Br J Haematol 1999 106 717 719 10.1046/j.1365-2141.1999.01615.x 10468863

[B11] Chng WJ Price-Troska T Gonzalea-Paz N Van Wier S Jacobus S Blood E Henderson K Oken M Van Ness B Greipp P Clinical significance of TP53 mutation in myeloma Leukemia 2007 21 582 584 10.1038/sj.leu.2404524 17215851

[B12] Chang H Qi C Yi Q Reece D Stewart AK p53 gene deletion detected by fluorescence in situ hybridization is an adverse prognostic factor for patients with multiple myeloma following autologous stem cell transplantation Blood 2005 105 358 360 10.1182/blood-2004-04-1363 15339849

[B13] Reece D Song KW Fu T Roland B Chang H Horsman DE Mansoor A Chen C Masih-Khan E Trieu Y Influence of cytogenetics in patients with relapsed or refractory multiple myeloma treated with lenalidomide plus dexamethasone: adverse effect of deletion 17p13 Blood 2009 114 522 525 10.1182/blood-2008-12-193458 19332768

[B14] Vogelstein B Lane D Levine AJ Surfing the p53 network Nature 2000 408 307 310 10.1038/35042675 11099028

[B15] Bates S Vousden KH Mechanisms of p53-mediated apoptosis Cell Mol Life Sci 1999 55 28 37 10.1007/s000180050267 10065149 PMC11147148

[B16] Ryan KM Ernst MK Rice NR Vousden KH Regulation and function of the p53 tumor suppressor protein Curr Opin Cell Biol 2001 13 332 337 10.1016/S0955-0674(00)00216-7 11343904

[B17] Stegh AH Targeting the p53 signaling pathway in cancer therapy - the promises, challenges and perils Expert Opin Ther Targets 2012 16 67 83 10.1517/14728222.2011.643299 22239435 PMC3291789

[B18] Essmann F Schulze-Osthoff K Translational approaches targeting the p53 pathway for anti-cancer therapy Br J Pharmacol 2012 165 328 344 10.1111/j.1476-5381.2011.01570.x 21718309 PMC3268188

[B19] Joerger AC Fersht AR The tumor suppressor p53: from structures to drug discovery Cold Spring Harb Perspect Biol 2010 2 a000919 10.1101/cshperspect.a000919 20516128 PMC2869527

[B20] Shangary S Wang S Small-molecule inhibitors of the MDM2-p53 protein-protein interaction to reactivate p53 function: a novel approach for cancer therapy Annu Rev Pharmacol Toxicol 2009 49 223 241 10.1146/annurev.pharmtox.48.113006.094723 18834305 PMC2676449

[B21] Selivanova G Therapeutic targeting of p53 by small molecules Semin Cancer Biol 2010 20 46 56 10.1016/j.semcancer.2010.02.006 20206268

[B22] Lane DP Brown CJ Verma C Cheok CF New insights into p53 based therapy Discov Med 2011 12 107 117 21878188

[B23] Wang W El-Deiry WS Restoration of p53 to limit tumor growth Curr Opin Oncol 2008 20 90 96 10.1097/CCO.0b013e3282f31d6f 18043262

[B24] Vassilev LT Vu BT Graves B Carvajal D Podlaski F Filipovic Z Kong N Kammlott U Lukacs C Klein C In vivo activation of the p53 pathway by small-molecule antagonists of MDM2 Science 2004 303 844 848 10.1126/science.1092472 14704432

[B25] Issaeva N Bozko P Enge M Protopopova M Verhoef LG Masucci M Pramanik A Selivanova G Small molecule RITA binds to p53, blocks p53-HDM-2 interaction and activates p53 function in tumors Nat Med 2004 10 1321 1328 10.1038/nm1146 15558054

[B26] Bykov VJ Issaeva N Shilov A Hultcrantz M Pugacheva E Chumakov P Bergman J Wiman KG Selivanova G Restoration of the tumor suppressor function to mutant p53 by a low-molecular-weight compound Nat Med 2002 8 282 288 10.1038/nm0302-282 11875500

[B27] Wiman KG Pharmacological reactivation of mutant p53: from protein structure to cancer patient Oncogene 2010 29 4245 4252 10.1038/onc.2010.188 20498645

[B28] Foster BA Coffey HA Morin MJ Rastinejad F Pharmacological rescue of mutant p53 conformation and function Science 1999 286 2507 2510 10.1126/science.286.5449.2507 10617466

[B29] Seemann S Maurici D Olivier M Caron de Fromentel C Hainaut P The tumor suppressor gene TP53: implications for cancer management and therapy Crit Rev Clin Lab Sci 2004 41 551 583 10.1080/10408360490504952 15603511

[B30] Saha MN Micallef J Qiu L Chang H Pharmacological activation of the p53 pathway in haematological malignancies J Clin Pathol 2010 63 204 209 10.1136/jcp.2009.070961 19955555

[B31] Tovar C Rosinski J Filipovic Z Higgins B Kolinsky K Hilton H Zhao X Vu BT Qing W Packman K Small-molecule MDM2 antagonists reveal aberrant p53 signaling in cancer: implications for therapy Proc Natl Acad Sci USA 2006 103 1888 1893 10.1073/pnas.0507493103 16443686 PMC1413632

[B32] Gu L Zhu N Findley HW Zhou M MDM2 antagonist nutlin-3 is a potent inducer of apoptosis in pediatric acute lymphoblastic leukemia cells with wild-type p53 and overexpression of MDM2 Leukemia 2008 22 730 739 10.1038/leu.2008.11 18273046 PMC3477706

[B33] Zhu N Gu L Li F Zhou M Inhibition of the Akt/survivin pathway synergizes the antileukemia effect of nutlin-3 in acute lymphoblastic leukemia cells Mol Cancer Ther 2008 7 1101 1109 10.1158/1535-7163.MCT-08-0179 18483299

[B34] Vilas-Zornoza A Agirre X Martín-Palanco V Martín-Subero JI San José-Eneriz E Garate L Álvarez S Miranda E Rodríguez-Otero P Rifón J Frequent and simultaneous epigenetic inactivation of TP53 pathway genes in acute lymphoblastic leukemia PLoS One 2011 6 e17012 10.1371/journal.pone.0017012 21386967 PMC3046174

[B35] Kojima K Konopleva M Samudio IJ Shikami M Cabreira-Hansen M McQueen T Ruvolo V Tsao T Zeng Z Vassilev LT MDM2 antagonists induce p53-dependent apoptosis in AML: implications for leukemia therapy Blood 2005 106 3150 3159 10.1182/blood-2005-02-0553 16014563 PMC1895324

[B36] Kojima K Konopleva M Samudio IJ Schober WD Bornmann WG Andreeff M Concomitant inhibition of MDM2 and Bcl-2 protein function synergistically induce mitochondrial apoptosis in AML Cell Cycle 2006 5 2778 2786 10.4161/cc.5.23.3520 17172851

[B37] Kojima K Konopleva M Samudio IJ Ruvolo V Andreeff M Mitogen-activated protein kinase kinase inhibition enhances nuclear proapoptotic function of p53 in acute myelogenous leukemia cells Cancer Res 2007 67 3210 3219 10.1158/0008-5472.CAN-06-2712 17409429

[B38] Zhang W Konopleva M Burks JK Dywer KC Schober WD Yang JY McQueen TJ Hung MC Andreeff M Blockade of mitogen-activated protein kinase/extracellular signal-regulated kinase kinase and murine double minute synergistically induces Apoptosis in acute myeloid leukemia via BH3-only proteins Puma and Bim Cancer Res 2010 70 2424 2434 10.1158/0008-5472.CAN-09-0878 20215498 PMC2840060

[B39] Secchiero P Zerbinati C di Iasio MG Melloni E Tiribelli M Grill V Zauli G Synergistic cytotoxic activity of recombinant TRAIL plus the non-genotoxic activator of the p53 pathway nutlin-3 in acute myeloid leukemia cells Curr Drug Metab 2007 8 395 403 10.2174/138920007780655432 17504227

[B40] Carter BZ Mak DH Schober WD Dietrich MF Pinilla C Vassilev LT Reed JC Andreeff M Triptolide sensitizes AML cells to TRAIL-induced apoptosis via decrease of XIAP and p53-mediated increase of DR5 Blood 2008 111 3742 3750 10.1182/blood-2007-05-091504 18187663 PMC2275030

[B41] Kojima K Konopleva M Tsao T Andreeff M Ishida H Shiotsu Y Jin L Tabe Y Nakakuma H Selective FLT3 inhibitor FI-700 neutralizes Mcl-1 and enhances p53-mediated apoptosis in AML cells with activating mutations of FLT3 through Mcl-1/Noxa axis Leukemia 2010 24 33 43 10.1038/leu.2009.212 19946262

[B42] Thompson T Andreeff M Studzinski GP Vassilev LT 1,25-dihydroxyvitamin D3 enhances the apoptotic activity of MDM2 antagonist nutlin-3a in acute myeloid leukemia cells expressing wild-type p53 Mol Cancer Ther 2010 9 1158 1168 10.1158/1535-7163.MCT-09-1036 20406950 PMC2868102

[B43] McCormack E Haaland I Venås G Forthun RB Huseby S Gausdal G Knappskog S Micklem DR Lorens JB Bruserud O Synergistic induction of p53 mediated apoptosis by valporic acid and nutlin-3 in acute myeloid leukemia Leukemia 2012 26 910 917 10.1038/leu.2011.315 22064349

[B44] Kojima K Shimanuki M Shikami M Samudio IJ Ruvolo V Corn P Hanaoka N Konopleva M Andreeff M Nakakuma H The dual PI3 kinase/mTOR inhibitor PI-103 prevents p53 induction by Mdm2 inhibition but enhances p53-mediated mitochondrial apoptosis in p53 wild-type AML Leukemia 2008 22 1728 1736 10.1038/leu.2008.158 18548093

[B45] Zauli G Celeghini C Melloni E Voltan R Ongari M Tiribelli M di Iasio MG Lanza F Secchiero P The sorafenib plus nutlin-3 combination promotes synergistic cytotoxicity in acute myeloid leukemic cells irrespectively of FLT3 and p53 status Haematologica 2012 97 1722 1730 10.3324/haematol.2012.062083 22689683 PMC3487447

[B46] Secchiero P Zerbinati C Melloni E Milani D Campioni D Fadda R Tiribelli M Zauli G The MDM-2 antagonist nutlin-3 promotes the maturation of acute myeloid leukemic blasts Neoplasia 2007 9 853 861 10.1593/neo.07523 17971905 PMC2040212

[B47] Lew QJ Tan CH Gurumurthy M Chu KL Cheong N Lane DP Chao SH NPMc(+) AML cell line shows differential protein expression and lower sensitivity to DNA-damaging and p53-inducing anticancer compounds Cell Cycle 2011 10 1978 1987 10.4161/cc.10.12.15859 21558800

[B48] Long J Parkin B Ouillette P Bixby D Shedden K Erba H Wang S Malek SN Multiple distinct molecular mechanisms influence sensitivity and resistance to MDM2 inhibitors in adult acute myelogenous leukemia Blood 2010 116 71 80 10.1182/blood-2010-01-261628 20404136 PMC2904583

[B49] Voltan R Celeghini C Melloni E Secchiero P Zauli G Perifosine plus nutlin-3 combination shows a synergistic anti-leukaemic activity Br J Haematol 2010 148 957 961 10.1111/j.1365-2141.2009.08018.x 19958355

[B50] Lu K Wang X Therapeutic advancement of chronic lymphocytic leukemia J Hematol Oncol 2012 5 55 10.1186/1756-8722-5-55 22980425 PMC3465197

[B51] Maddocks KJ Lin TS Update in the management of chronic lymphocytic leukemia J Hematol Oncol 2009 2 29 10.1186/1756-8722-2-29 19619273 PMC2723130

[B52] Secchiero P di Iasio MG Melloni E Voltan R Celeghini C Tiribelli M Dal Bo M Gattei V Zauli G The expression levels of the pro-apoptotic XAF-1 gene modulate the cytotoxic response to Nutlin-3 in B chronic lymphocytic leukemia Leukemia 2010 24 480 483 10.1038/leu.2009.215 19847196

[B53] Secchiero P Melloni E Tiribelli M Gonelli A Zauli G Combined treatment of CpG-oligodeoxynucleotide with Nutlin-3 induces strong immune stimulation coupled to cytotoxicity in B-chronic lymphocytic leukemic (B-CLL) cells J Leukoc Biol 2008 83 434 437 17998303 10.1189/jlb.0707459

[B54] Secchiero P Barbarotto E Tiribelli M Zerbinati C di Lasio MG Gonelli A Cavazzini F Campioni D Fanin R Cuneo A Functional integrity of the p53-mediated apoptotic pathway induced by the nongenotoxic agent nutlin-3 in B-cell chronic lymphocytic leukemia (B-CLL) Blood 2006 107 4122 4129 10.1182/blood-2005-11-4465 16439677

[B55] Coll-Mulet L Iglesias-Serret D Santidrian AF Cosialls AM de Frias M Castano E Campàs C Barragán M de Sevilla AF Domingo A MDM2 antagonists activate p53 and synergize with genotoxic drugs in B-cell chronic lymphocytic leukemia cells Blood 2006 107 4109 4114 10.1182/blood-2005-08-3273 16439685

[B56] Zauli G di Iasio MG Secchiero P Dal Bo M Marconi D Bomben R Del Poeta G Gattei V Exposure of B cell chronic lymphocytic leukemia (B-CLL) cells to nutlin-3 induces a characteristic gene expression profile, which correlates with nutlin-3-mediated cytotoxicity Curr Cancer Drug Targets 2009 9 510 518 10.2174/156800909788486777 19519319

[B57] Bo MD Secchiero P Degan M Marconi D Bomben R Pozzato G Gaidano G Del Poeta G Forconi F Zauli G Gattei V MDM4 (MDMX) is overexpressed in chronic lymphocytic leukaemia (CLL) and marks a subset of p53wild-type CLL with a poor cytotoxic response to Nutlin-3 Br J Haematol 2010 150 237 239 20507307 10.1111/j.1365-2141.2010.08185.x

[B58] Kojima K Konopleva M McQueen T O’Brien S Plunkett W Andreeff M Mdm2 inhibitor Nutlin-3a induces p53-mediated apoptosis by transcription-dependent and transcription-independent mechanisms and may overcome Atm-mediated resistance to fludarabine in chronic lymphocytic leukemia Blood 2006 108 993 1000 10.1182/blood-2005-12-5148 16543464 PMC1895860

[B59] Zauli G Voltan R Bosco R Melloni E Marmiroli S Rigolin GM Cuneo A Secchiero P Dasatinib plus Nutlin-3 shows synergistic antileukemic activity in both p53 wild-type and p53 mutated B chronic lymphocytic leukemias by inhibiting the Akt pathway Clin Cancer Res 2011 17 762 770 10.1158/1078-0432.CCR-10-2572 21106726

[B60] Steele AJ Prentice AG Hoffbrand AV Yogashangary BC Hart SM Nacheva EP Howard-Reeves JD Duke VM Kottaridis PD Cwynarski K p53-mediated apoptosis of CLL cells: evidence for a transcription-independent mechanism Blood 2008 112 3827 3834 10.1182/blood-2008-05-156380 18682598

[B61] Peterson LF Mitrikeska E Giannola D Lui Y Sun H Bixby D Malek SN Donato NJ Wang S Talpaz M p53 stabilization induces apoptosis in chronic myeloid leukemia blast crisis cells Leukemia 2011 25 761 769 10.1038/leu.2011.7 21350558

[B62] Kurosu T Wu N Oshikawa G Kagechika H Miura O Enhancement of imatinib-induced apoptosis of BCR/ABL-expressing cells by nutlin-3 through synergistic activation of the mitochondrial apoptotic pathway Apoptosis 2010 15 608 620 10.1007/s10495-010-0457-0 20094798

[B63] Stuhmer T Chatterjee M Hildebrandt M Herrmann P Gollasch H Gerecke C Theurich S Cigliano L Manz RA Daniel PT Nongenotoxic activation of the p53 pathway as a therapeutic strategy for multiple myeloma Blood 2005 106 3609 3617 10.1182/blood-2005-04-1489 16081689

[B64] Ooi MG Hayden PJ Kotoula V McMillin DW Charalambous E Daskalaki E Raje NS Munshi NC Chauhan D Hideshima T Interactions of the Hdm2/p53 and proteasome pathways may enhance the antitumor activity of bortezomib Clin Cancer Res 2009 15 7153 7160 10.1158/1078-0432.CCR-09-1071 19934289 PMC3672410

[B65] Saha MN Jiang H Chang H Molecular mechanisms of nutlin-induced apoptosis in multiple myeloma: evidence for p53-transcription-dependent and -independent pathways Cancer Biol Ther 2010 10 567 578 10.4161/cbt.10.6.12535 20595817 PMC3040946

[B66] Zhang Q Lu H Nutlin’s two roads toward apoptosis Cancer Biol Ther 2010 10 579 581 10.4161/cbt.10.6.13127 20716967 PMC3040947

[B67] Drakos E Thomaides A Medeiros LJ Li J Leventaki V Konopleva M Andreeff M Rassidakis GZ Inhibition of p53-murine double minute 2 interaction by nutlin stabilizes p53 and induces cell cycle arrest and apoptosis in Hodgkin lymphoma Clin Cancer Res 2007 13 3380 3387 10.1158/1078-0432.CCR-06-2581 17545546

[B68] Janz M Stühmer T Vassilev LT Bargou RC Pharmacologic activation of p53-dependent and p53-independent apoptotic pathways in Hodgkin/Reed-Sternberg cells Leukemia 2007 21 772 779 17268519 10.1038/sj.leu.2404565

[B69] Jones RJ Baladandayuthapani V Neelapu S Fayad LE Romaguera JE Wang M Sharma R Yang D Orlowski RZ HDM-2 inhibition suppresses expression of ribonucleotide reductase subunit M2, and synergistically enhances gemcitabine-induced cytotoxicity in mantle cell lymphoma Blood 2011 118 4140 4149 10.1182/blood-2011-03-340323 21844567 PMC3204731

[B70] Drakos E Atsaves V Li J Leventaki V Andreeff M Medeiros LJ Rassidakis GZ Stabilization and activation of p53 downregulates mTOR signaling through AMPK in mantle cell lymphoma Leukemia 2009 23 784 790 10.1038/leu.2008.348 19225536

[B71] Jin L Tabe Y Kojima K Zhou Y Pittaluga S Konopleva M Miida T Raffeld M MDM2 antagonist Nutlin-3 enhances bortezomib-mediated mitochondrial apoptosis in TP53-mutated mantle cell lymphoma Cancer Lett 2010 299 161 170 10.1016/j.canlet.2010.08.015 20850924

[B72] Tabe Y Sebasigari D Jin L Rudelius M Davies-Hill T Miyake K Miida T Pittaluga S Raffeld M MDM2 antagonist nutlin-3 displays antiproliferative and proapoptotic activity in mantle cell lymphoma Clin Cancer Res 2009 15 933 942 10.1158/1078-0432.CCR-08-0399 19188164 PMC7322626

[B73] Drakos E Atsaves V Schlette E Li J Papanastasi I Rassidakis GZ Medeiros LJ The therapeutic potential of p53 reactivation by nutlin-3a in ALK + anaplastic large cell lymphoma with wild-type or mutated p53 Leukemia 2009 23 2290 2299 10.1038/leu.2009.180 19741726

[B74] Sosin AM Burger AM Siddiqi A Abrams J Mohammad RM Al-Katib AM HDM2 antagonist MI-219 (spiro-oxindole), but not Nutlin-3 (cis-imidazoline), regulates p53 through enhanced HDM2 autoubiquitination and degradation in human malignant B-cell lymphomas J Hematol Oncol 2012 5 57 10.1186/1756-8722-5-57 22989009 PMC3473265

[B75] Drakos E Singh RR Rassidakis GZ Schlette E Li J Claret FX Ford RJ Jr Vega F Medeiros LJ Activation of the p53 pathway by the MDM2 inhibitor nutlin-3a overcomes BCL2 overexpression in a preclinical model of diffuse large B-cell lymphoma associated with t(14;18)(q32;q21) Leukemia 2011 25 856 867 10.1038/leu.2011.28 21394100 PMC3094765

[B76] Renouf B Hollville E Pujals A Tétaud C Garibal J Wiels J Activation of p53 by MDM2 antagonists has differential apoptotic effects on Epstein-Barr virus (EBV)-positive and EBV-negative Burkitt’s lymphoma cells Leukemia 2009 23 1557 1563 10.1038/leu.2009.92 19421231

[B77] Tageja N Padheye S Dandawate P Al-Katib A Mohammad RM New targets for the treatment of follicular lymphoma J Hematol Oncol 2009 2 50 10.1186/1756-8722-2-50 20030851 PMC2805680

[B78] Mohammad RM Wu J Azmi AS Aboukameel A Sosin A Wu S Yang D Wang S Al-Katib AM An MDM2 antagonist (MI-319) restores p53 functions and increases the life span of orally treated follicular lymphoma bearing animals Mol Cancer 2009 8 115 10.1186/1476-4598-8-115 19958544 PMC2794250

[B79] Hasegawa H Yamada Y Iha H Tsukasaki K Nagai K Atogami S Sugahara K Tsuruda K Ishizaki A Kamihira S Activation of p53 by Nutlin-3a, an antagonist of MDM2, induces apoptosis and cellular senescence in adult T-cell leukemia cells Leukemia 2009 23 2090 2101 10.1038/leu.2009.171 19710698

[B80] Enge M Bao W Hedström E Jackson SP Moumen A Selivanova G MDM2-dependent downregulation of p21 and hnRNP K provides a switch between apoptosis and growth arrest induced by pharmacologically activated p53 Cancer Cell 2009 15 171 183 10.1016/j.ccr.2009.01.019 19249676

[B81] Jones RJ Bjorklund CC Baladandayuthapani V Kuhn DJ Orlowski RZ Drug Resistance to Inhibitors of the Human Double Minute-2 E3 Ligase Is Mediated by Point Mutations of p53, but Can Be Overcome with the p53 Targeting Agent RITA Mol Cancer Ther 2012 11 2243 2253 10.1158/1535-7163.MCT-12-0135 22933706 PMC3469746

[B82] Kazemi A Safa M Shahbazi A RITA enhances chemosensivity of pre-B ALL cells to doxorubicin by inducing p53-dependent apoptosis Hematology 2011 16 225 231 10.1179/102453311X12953015767536 21756539

[B83] Saha MN Jiang H Muaki A Chang H RITA inhibits multiple myeloma cell growth through induction of p53-mediated caspase-dependent apoptosis and synergistically enhances nutlin-induced cytotoxic responses Mol Cancer Ther 2010 9 3041 3051 10.1158/1535-7163.MCT-10-0471 21062913

[B84] Saha MN Jiang H Yang Y Zhu X Wang X Schimmer AD Chang H RITA-induced apoptosis of multiple myeloma cells is mediated by activation of JNK signaling Blood (ASH Annual Meeting Abstracts) 2011 118 1836

[B85] Saha MN Yang Y Chang H Targeting p53 by small molecule p53 activators in multiple myeloma [abstract] J Hematol Oncol 2012 5 Suppl 1 A7

[B86] Krajewski M Ozdowy P D’Silva L Rothweiler U Holak TA NMR indicates that the small molecule RITA does not block p53-MDM2 binding in vitro Nat Med 2005 11 1135 1136 10.1038/nm1105-1135 16270059

[B87] Saha MN Jiang H Yang Y Zhu X Wang X Schimmer AD Qiu L Chang H Targeting p53 via JNK Pathway: A Novel Role of RITA for Apoptotic Signaling in Multiple Myeloma PLoS One 2012 7 e30215 10.1371/journal.pone.0030215 22276160 PMC3262803

[B88] Nahi H Merup M Lehmann S Bengtzen S Möllgård L Selivanova G Wiman KG Paul C PRIMA-1 induces apoptosis in acute myeloid leukaemia cells with p53 gene deletion Br J Haematol 2006 132 230 236 10.1111/j.1365-2141.2005.05851.x 16398657

[B89] Lambert JM Gorzov P Veprintsev DB Söderqvist M Segerbäck D Bergman J Fersht AR Hainaut P Wiman KG Bykov VJ PRIMA-1 reactivates mutant p53 by covalent binding to the core domain Cancer Cell 2009 15 376 388 10.1016/j.ccr.2009.03.003 19411067

[B90] Bykov VJ Issaeva N Selivanova G Wiman KG Mutant p53-dependent growth suppression distinguishes PRIMA-1 from known anticancer drugs: a statistical analysis of information in the National Cancer Institute database Carcinogenesis 2002 23 2011 2018 10.1093/carcin/23.12.2011 12507923

[B91] Lehmann S Bykov VJ Ali D Andrén O Cherif H Tidefelt U Uggla B Yachnin J Juliusson G Moshfegh A Targeting p53 in vivo: A first-in-human study with p53-targeting compound APR-246 in refractory hematologic malignancies and prostate cancer J Clin Oncol 2012 30 3633 3639 10.1200/JCO.2011.40.7783 22965953

[B92] Nahi H Lehmann S Mollgard L Bengtzen S Selivanova G Wiman KG Paul C Merup M Effects of PRIMA-1 on chronic lymphocytic leukaemia cells with and without hemizygous p53 deletion Br J Haematol 2004 127 285 291 10.1111/j.1365-2141.2004.05210.x 15491287

[B93] Ali D Jönsson-Videsäter K Deneberg S Bengtzén S Nahi H Paul C Lehmann S APR-246 exhibits anti-leukemic activity and synergism with conventional chemotherapeutic drugs in acute myeloid leukemia cells Eur J Haematol 2011 86 206 215 10.1111/j.1600-0609.2010.01557.x 21114538

[B94] Saha MN Jiang H Mei-His C Chang H p53-independent anti-myeloma activity of Prima-1^met^ Blood (ASH Annual Meeting Abstracts) 2011 118 1826

[B95] Bykov VJ Issaeva N Zache N Shilov A Hultcrantz M Bergman J Selivanova G Wiman KG Reactivation of mutant p53 and induction of apoptosis in human tumor cells by maleimide analogs J Biol Chem 2005 280 30384 30391 10.1074/jbc.M501664200 15998635

[B96] Saha MN Jiang H Chang H A novel small molecule MIRA-1 induces cytotoxicity in multiple myeloma cells harbouring wild type or mutant p53 Modern Pathology 2012 25 (Suppl 2) 153422766788

[B97] Leiba M Jakubikova J Klippel S Mitsiades CS Hideshima T Tai YT Leiba A Pines M Richardson PG Nagler A Anderson KC Halofuginone inhibits multiple myeloma growth in vitro and in vivo and enhances cytotoxicity of conventional and novel agents Br J Haematol 2012 157 718 731 10.1111/j.1365-2141.2012.09120.x 22533681 PMC4414398

[B98] Kojima K Duvvuri S Ruvolo V Samaniego F Younes A Andreeff M Decreased sensitivity of 17p-deleted chronic lymphocytic leukemia cells to a small molecule BCL-2 antagonist ABT-737 Cancer 2012 118 1023 1031 10.1002/cncr.26360 21761401 PMC3579224

[B99] Kojima K Burks JK Arts J Andreeff M The novel tryptamine derivative JNJ-26854165 induces wild-type p53- and E2F1-mediated apoptosis in acute myeloid and lymphoid leukemias Mol Cancer Ther 2010 9 2545 2557 10.1158/1535-7163.MCT-10-0337 20736344 PMC2949269

[B100] Görgün G Calabrese E Hideshima T Ecsedy J Perrone G Mani M Ikeda H Bianchi G Hu Y Cirstea D A novel Aurora-A kinase inhibitor MLN8237 induces cytotoxicity and cell-cycle arrest in multiple myeloma Blood 2010 115 5202 5213 10.1182/blood-2009-12-259523 20382844 PMC2892955

[B101] Wang S Zhao Y Bernard D Aguilar A Kumar S Targeting the MDM2-p53 protein-protein interaction for new cancer therapeutics Top Med Chem 2012 8 57 80 10.1007/978-3-642-28965-1_2

[B102] Saha MN Jiang H Jayakar J Reece D Branch DR Chang H MDM2 antagonist nutlin plus proteasome inhibitor velcade combination displays a synergistic anti-myeloma activity Cancer Biol Ther 2010 9 936 944 10.4161/cbt.9.11.11882 20418664

[B103] Surget S Chiron D Gomez-Bougie P Descamps G Ménoret E Bataille R Moreau P Le Gouill S Amiot M Pellat-Deceunynck C Cell death via DR5, but not DR4, is regulated by p53 in myeloma cells Cancer Res 2012 72 4562 4573 10.1158/0008-5472.CAN-12-0487 22738917

[B104] Ambrosini G Sambol EB Carvajal D Vassilev LT Singer S Schwartz GK Mouse double minute antagonist Nutlin-3a enhances chemotherapy-induced apoptosis in cancer cells with mutant p53 by activating E2F1 Oncogene 2007 26 3473 3481 10.1038/sj.onc.1210136 17146434

[B105] Lau LM Nugent JK Zhao X Irwin MS HDM2 antagonist Nutlin-3 disrupts p73-HDM2 binding and enhances p73 function Oncogene 2008 27 997 1003 10.1038/sj.onc.1210707 17700533

[B106] Kravchenko JE Ilyinskaya GV Komarov PG Agapova LS Kochetkov DV Strom E Frolova EI Kovriga I Gudkov AV Feinstein E Small-molecule RETRA suppresses mutant p53-bearing cancer cells through a p73-dependent salvage pathway Proc Natl Acad Sci USA 2008 105 6302 6307 10.1073/pnas.0802091105 18424558 PMC2327210

[B107] Alsafadi S Tourpin S André F Vassal G Ahomadegbe JC p53 family: at the crossroads in cancer therapy Curr Med Chem 2009 16 4328 4344 10.2174/092986709789578196 19754415

